# Leveraging a large language model to support expansion of surveillance activities to include cardiovascular implantable device infections in a large, integrated national healthcare system

**DOI:** 10.1017/ice.2025.10384

**Published:** 2026-04

**Authors:** Dipandita Basnet, Hillary J. Mull, Daniel J. Morgan, Samuel W. Golenbock, Rebecca P. Lamkin, Judith M. Strymish, Kimberly Harvey, Kaeli Yuen, Marin L. Schweizer, Dimitri Drekonja, Maria C. Rodriguez-Barradas, Westyn Branch-Elliman

**Affiliations:** 1 Center for Health Optimization and Implementation Research (CHOIR), VA Boston Healthcare System, Boston, MA, USA; 2 Boston University School of Public Health, Boston, MA, USA; 3 Department of Surgery, Boston University Chobanian & Avedisian School of Medicine, Boston, MA, USA; 4 VA Maryland Healthcare System, Baltimore, MD, USA; 5 Department of Epidemiology and Public Health, University of Maryland School of Medicine, Baltimore, MD, USA; 6 Section of Infectious Disease, VA Boston Healthcare System, Boston, MA, USA; 7 Harvard Medical School, Boston, MA, USA; 8 Department of Veterans Affairs, Office of the Chief AI Officer, Washington, DC, USA; 9 William S. Middleton VA Hospital, Madison, WI, USA; 10 University of Wisconsin-Madison, Madison, WI, USA; 11 Section of Infectious Diseases, Minneapolis VA Healthcare System, MN, USA; 12 Section of Infectious Diseases, Michael E. DeBakey VA Medical Center, Houston, TX, USA; 13 Department of Medicine, Baylor College of Medicine, Houston, TX, USA; 14 Section of Infectious Diseases, VA Greater Los Angeles Healthcare System, Los Angeles, CA, USA; 15 Department of Medicine, UCLA David Geffen School of Medicine, Los Angeles, CA, USA; 16 Center for the Study of Healthcare Innovation, Implementation, and Policy, Greater Los Angeles VA Medical Center, Los Angeles, CA, USA

## Abstract

**Background:**

Surveillance activities are emerging as exemplar use cases for large language models (LLMs) in health care. The aim of this study was to evaluate the potential for LLMs to support the expansion of surveillance activities to include cardiovascular implantable electronic device (CIED) procedures.

**Methods:**

A validated machine learning-based infection flagging tool was applied to a cohort of VA CIED procedures from 7/1/2021 to 9/30/2023; cases with ≥10% probability of CIED infection underwent manual review. Then, a weighted random sample of 50 infected and 50 uninfected cases was reviewed with generative artificial intelligence (GenAI) assistance. GenAI prompts were iteratively refined to extract and classify all components of infection-related variables from clinical notes. Data extracted by GenAI were compared with manual chart reviews to assess infection status and extraction consistency.

**Results:**

Among 12,927 CIED procedures, 334 (2.58%) had ≥10% probability of CIED infection. Among 100 sampled cases, 50 of 50 uninfected cases were correctly categorized. Among 50 infection cases, GenAI identified all CIED infections, but the timing of events and the attribution to a preceding procedure were incorrect in 7 of 50 cases. The overall specificity of the GenAI-assisted process was 100% and the sensitivity for accurately classifying timing and attribution of CIED infection events was 82%. Errors in timing improved with iterative prompt updates. Manual chart reviews averaged 25 minutes per chart; the GenAI-assisted process averaged 5–7 minutes per chart.

**Conclusions:**

LLMs can help streamline the review process for healthcare-associated infection surveillance, but manual adjudication of output is needed to ensure the correct timeline of events and attribution.

## Background

Mandatory healthcare-associated infection (HAI) surveillance activities are emerging as an exemplar use case for the application of generative artificial intelligence (GenAI) in health care. Previous work applied GenAI for central line-associated bloodstream infections (CLABSI) and catheter-associated urinary tract infections (CAUTI) surveillance in limited, retrospective settings and/or on synthetic datasets.^1–5^ These proof-of-concept studies show promise, although ongoing challenges include limited access to the full range of data available in the electronic health record (EHR).^1^ Prior work does not address real-world challenges in operationalizing these tools.^5,6^ Curated datasets are inherently filtered and direct the large language models (LLMs) to the important information; the tools therefore often do not face the challenge of primarily finding information in the EHR and handling complex and often contradictory information. Additionally, LLMs are complex models that require substantial computing infrastructure to be run and applied to the entire corpus of data available in the EHR.^7–10^ Even if the computing infrastructure is available, simply running these models on complete healthcare datasets can take substantial time depending on cohort size and the health system. Potential solutions to address computing power and complexity include expanding cloud capacity, applying more specific models, and identifying ways to curate the dataset such that the LLMs can be directed toward a smaller corpus of unstructured data.^11^ Although expanding infrastructure is costly, reducing the unstructured dataset size and directing the GenAI tool to a curated sample with higher true-event probability can enhance feasibility. Real-world experiences demonstrating the feasibility and accuracy of this phased approach to directing the GenAI to only the highest probability cases where detailed review is needed are limited.

**Figure 1.**
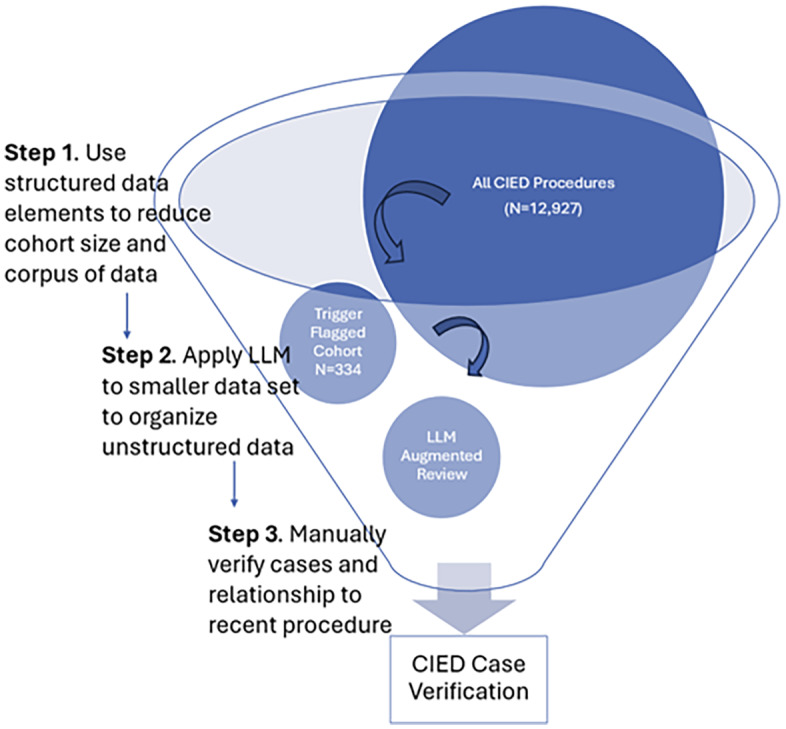
Hybrid process for directing GenAI tools to support sustainable scaling for healthcare-associated infection surveillance. GenAI systems have substantial computing infrastructure requirements that limit the feasibility of scaling in healthcare systems. A phased approach, in which less computationally complex algorithms are first applied to direct the GenAI tools to the highest probability cases and then GenAI tools are applied to a smaller, more curated dataset, may provide a pathway to feasible and sustainable deployment of GenAI tools for healthcare applications, such as healthcare-associated infection surveillance.

Our group previously developed, validated, and implemented a trigger-tool-based mechanism to support the expansion of HAI surveillance to include cardiovascular implantable electronic device (CIED) infections.^3,12^ The machine learning-based surveillance algorithm uses a combination of structured variables and text note searches to flag potential infection cases.^3,12^ After real-world testing and updates to include community care (CC) data, the surveillance algorithm flagged 2.58% of cases and had a positive predictive value (PPV) of 74.0%.^3^ Although the PPV is insufficiently high to leverage the surveillance algorithm as a stand-alone HAI reporting tool without additional manual review in most settings, the surveillance algorithm was highly effective at ruling out cases with negative infection, effectively reducing the size of the cohort requiring manual review to confirm the presence of CIED infection from 12,927 to 334.^3^ We hypothesized that LLMs newly available in the VA healthcare system, specifically GPT-4o accessed through Azure OpenAI, could be integrated into the surveillance workflow to more fully automate the process. Thus, the aim of this study was to evaluate the accuracy of GenAI for performing the second stages of CIED infection review, including assessments of each element of the infection determination process, following a reduction in cohort size using less computationally complex informatics strategies (Figure 1).

## Methods

### Cohort creation

Details of the creation of the retrospective cohort used to conduct this work are previously described.^3,12,13^ Briefly, CIED procedures conducted in the VA healthcare system from 7/2021–9/2023 were identified using Current Procedural Terminology (CPT) codes. The previously developed machine learning algorithm for flagging CIED procedures with a high probability of infection (>10%)^3,13^ was then applied, and all cases underwent manual review to adjudicate the presence or absence of a procedure-related CIED infection. The machine learning tool includes VA clinical data, including text note flags from clinical notes, as well as Community Care data. Community care data includes admissions, CPT codes, and medication dispensing data but not clinical note information.^3,13^ To test the quality of the GenAI-assisted chart review process, a stratified random sample of the manually reviewed dataset with CIED infection (N = 50) and without CIED infection (N = 50) underwent a second round of review using GenAI (VA GPT 4.0). The stratified random sampling strategy was used to ensure that a diversity of VA sites were included and the sample size was based on the feasibility of a second manual review and previously published studies using similar approaches.

### CIED infection definitions

CIED infection status was determined based on the presence of at least 2 symptoms (eg, erythema, pain, swelling, or drainage/pus at the insertion site, fever, chills, sweats), and/or echocardiographic evidence of infection (lead involvement or valve involvement/endocarditis), and/or positive wound site culture or blood cultures with directed treatment and/or clinician diagnosis of infection (see Supplementary Material 1 for chart review tool).^13^ Infection diagnosis required a minimum of 2 symptoms plus treatment or a clinician diagnosis. Cases with possibly compatible symptoms and a clear alternative diagnosis (eg, pain and swelling and hematoma diagnosis) were not classified as infections. Any clinician diagnosis with treatment was classified as an infection. Pocket infections were defined as infections confined to the battery pocket/wound site; systemic/lead infections required evidence of systemic infection with either positive blood cultures or echocardiographic evidence of infection. Aligned with Centers for Disease Control and Prevention surgical site infection (SSI) definitions with device placement, CIED infections that occurred within 90 days of a procedure and that were not existing at the time of the procedure were considered to be procedure-related.^14^ Infections that occurred outside of the 90-day window and procedures that were performed for managing an existing infection (eg, the infection predated the procedure) were classified as not procedure-related.

### Development of prompts and GenAI infection determination process

VA GenAI is a generative AI chat interface program fully contained within the VA ecosystem that does not retain patient data. VA GenAI leverages GPT-4o, accessed through Azure OpenAI, with a temperature set to zero. Due to the closed nature of the VA healthcare informatics ecosystem and the lack of integration with VISTA, relevant clinical notes were manually pulled and entered into the GenAI system. Time to complete the note harvesting and the copy-paste effort were tracked to compare with the original manual review process.

Based on prior experience with a GenAI prompt to identify CLABSIs and a previously developed CIED infection chart abstraction tool, an initial GenAI prompt to measure relevant variables for the CIED infection determination was created.^1^ GenAI-assisted chart review process began with aggregating clinical notes from the Joint Legacy Viewer into the GenAI platform as a single, continuous document. The GenAI process was designed to be two phased: first, to organize the data elements and, second, to summarize the information and make a determination about whether a CIED infection was present and if it occurred in the 90-day surveillance window (eg, was procedure-related). For the first phase (data extraction and organization), prompts were designed to provide a comprehensive summary of the key pieces of information included in the chart review tool needed to make a determination about the presence of CIED infection, for example, identify symptoms, microbiologic evidence, etc. Then, the GenAI tool was asked to apply the same CIED infection definitions and National Healthcare Safety Network (NHSN) criteria about infection timing used for the manual review to make a final determination about CIED infection presence and HAI attribution.

The GenAI prompts were iteratively updated until they reliably organized the relevant information needed to populate the chart review tool and make an infection determination (Table 1, Supplementary Material 2). Following prompt development, clinical data cut and pasted from EHR notes were provided to the VA GenAI tool along with the iteratively updated and improved prompts.

**Table 1.** Prompt revisions for accurate cardiovascular implantable electronic device (CIED) infection date extraction and attribution as procedure-related from GPT-4o

Prompt version	Prompt text	Generated response/errors	Notes for revision
**Initial prompt**	What is the date of the CIED infection?	• The date of the CIED infection is between December 26 and 29, 2021.	Add more details to the prompt to specify which date to select in case of multiple dates provided.
**Revision 1**	What is the date of the CIED infection? Please determine the date of the CIED infection based on the clinical notes provided. If multiple dates are mentioned, select the most recent one associated with the infection symptoms. The infection date is the earlier of either the initial presentation date or the first positive microbiologic culture date.	• Initial Presentation Date/Admission Date: December 27, 2021. • First Positive Microbiologic Culture Date: December 28, 2021 (Blood culture positive for Gram-negative rods)	Ensure the prompt guides to select the most accurate date based on the provided criteria. Further instructions might be needed to avoid confusion in cases with close date ranges
**Revision 2, final prompt**	Identifying the date of CIED infection Context: You are provided with clinical notes regarding a patient who has a Cardiovascular Implantable Electronic Device (CIED). Your task is to accurately identify the date of the CIED infection. Instructions: Look through the clinical notes to identify and extract the date associated with the CIED infection. Consider infection-related keywords: Focus on mentions of infection-related terms such as “infection,” “erythema,” “tenderness,” “pus,” “swelling,” “fever,” “systemic signs,” and results from diagnostic tests like echocardiography or culture reports. If multiple dates are mentioned, select the date most directly associated with the onset or diagnosis of the infection symptoms. The infection date is the earlier of either the initial presentation date or the first positive microbiologic culture date. Date of CIED infection: What is the most accurate date corresponding to the CIED infection based on the clinical notes?	• The most accurate date corresponding to the CIED infection, based on the clinical notes provided, is December 27, 2021. • This date is identified as the initial presentation date when the patient was admitted with evidence of a pacemaker lead infection and associated symptoms • The patient presented with fever, drainage, and the pacemaker lead protruding from the skin. • The first positive microbiologic culture indicating Gram-negative rods (later identified as Pseudomonas) was obtained on December 28, 2021. • Therefore, December 27, 2021, marks the onset or initial presentation date of the infection, establishing it as the accurate date corresponding to the CIED infection. *No errors encountered in the response.	Final prompt is detailed and accurate, providing clear instructions on how to identify and report the date.

### Analysis: GenAI-alone versus manual review alone

GenAI summary determination (infection yes/no) and accuracy of each of the individual elements were evaluated with manual review determination used as the standard of comparison. Sensitivity and specificity of the GenAI determination versus manual review were calculated and described using simple descriptive statistics; sensitivity and specificity are presented for final, refined prompts only. Additionally, the time taken for each stage, from EHR note extraction to Excel data entry, was quantitatively measured. The qualitative assessment included classifying errors, categorizing them by type, and evaluating prompt quality and updates. Variables extracted by GenAI were compared to manual determinations, with discrepancies and errors recorded and categorized for further evaluation.

### Ethical considerations

This study was approved as exempt human subjects research by the VA Boston Research and Development Committee prior to data collection and analysis. VA GPT is Health Insurance Portability and Accountability Act (HIPAA)-compliant and does not store user data or retain data input into the interface. It is authorized to capture and handle Protected Health Information, Personally Identifiable Information, and other VA sensitive data securely.

## Results

During the study period, 12,927 CIED procedures were performed, and 334 (2.58%) were flagged by the algorithm. This included confirmed infection cases (n = 50) and confirmed non-infection cases (n = 50) from 71 VA medical centers; among the 50 CIED infection cases, 46 occurred during the 90-day surveillance window period following the index procedure, and 4 were preexisting infections present at the time of the procedure. Patients in the reviewed sample were predominantly male (98%) and non-Hispanic white (61%) with an average age of 74.2 years (standard deviation, 10.5 years). The CIED infections included 31 pocket infections, 9 cases of systemic infections and endocarditis, 7 cases of lead infection, 1 case of cellulitis, 1 case of left ventricular assist device infection, and 1 infection with evidence of both pocket involvement and lead infection (mixed case). Most CIED infection cases had positive blood cultures (37 cases) and/or wound cultures (4 cases). Blood culture isolates included *Staphylococcus aureus* (11), *Escherichia coli* (2), Streptococcal species (2), and others (22). Wound culture isolates included *S. aureus* (2), *Serratia marcescens* (1), and *Proteus Mirabilis* (1). The accuracy of each element in the CIED infection review process is presented in Table 2. GenAI correctly identified CIED infection presence in 50 of 50 true infection cases. The most common errors were related to the accurate ascertainment of dates and timing of events. Initially, GenAI correctly identified 43 of 50 procedure dates and 49 of 50 culture collection dates. Accuracy of the procedure date was able to be improved with iterative prompt updates (Table 1); the culture date error was not able to be corrected because the culture was collected outside of the VA healthcare system and that information was not available to the GenAI tool. Both methods accurately identified infection-related symptoms, clinical diagnoses, procedural interventions, and antibiotic treatments in all 50 infection cases. Through iterative prompt engineering focusing on infection-related keywords and specific instructions about how to assess infection onset, GenAI’s performance for identifying dates correctly improved substantially (See Supplementary Material 3 for exemplar output).

**Table 2.** Accuracy of individual data elements and data organization process conducted by GenAI versus manual review

Data element	GenAI accuracy*	Reason for discordance between GenAI output and manual review
Overall CIED infection yes/no determination	50/50 (100%)	N/A
Date of procedure	43/50 (86%)	The initial prompt provided to GenAI did not perfectly achieve the desired output. After iterative updates to the prompt, all 7 cases accurately provided the exact date of the procedure.
Procedure-related infection (eg, after procedure but within 90-day window)	39/46 (85%)	GenAI was unable to create appropriate timeline.
Preexisting infection (outside of the 90-day window and procedures that were performed for managing an existing infection)	4/4 (100%)	N/A
CIED clinical symptoms (eg, fever, erythema)	50/50 (100%)	N/A
Clinician diagnosis of infection	50/50 (100%)	N/A
Type of infection	49/50 (98%)	Evidence of a mixed infection (eg, evidence of both local and systemic infection)
Clinical culture date	49/50 (98%)	Cultures not done at the VA
Procedural intervention	50/50 (100%)	N/A
Antibiotic therapy	50/50 (100%)	N/A

GenAI, generative artificial intelligence; CIED, cardiovascular implantable electronic device.

* Manual review process used as the standard of comparison.

GenAI was generally able to summarize clinical information to determine infection severity, correctly ascertaining local versus systemic infection in 49 of 50 cases; the one misclassification was due to a mixed infection with evidence of both pocket and systemic involvement. In this case, the GenAI classified the infection type in the following manner:

“CIED infection-present… While there are indications suggestive of a systemic infection, the specific involvement of the device pocket or definitive imaging results for endocarditis were not provided. Based on the clinical information provided it could either be a pocket infection or endocarditis. Further imaging studies and clinical evaluations are necessary to accurately determine the precise nature of the infection.”

After data extraction and organization, the GenAI system was able to summarize the information and correctly identify all 50 CIED infection cases as having an infection and correctly ruled out infection in all 50 uninfected cases. Of the 46 procedure-related infections, 39 were correctly attributed. Most errors were due to failures to identify dates and the timeline of events (Table 2). GenAI correctly created timelines in 42 of 50 cases and 49 of 50 infection types, resulting in 82% sensitivity (95% CI: 68.6%–91.4%) and 100% specificity (95% CI: 92.9%–100%) (Table 3).

**Table 3.** Accuracy of the generative artificial intelligence (GenAI) tool summary for measuring procedure-attributable cardiovascular implantable electronic device infections

	**Manual review of infection**				
**GenAI determination of infection**	**Manual yes**	**Manual no**	**Total**	**Sensitivity (95% CI)**	**Specificity (95% CI)**
**GenAI yes**	41	0	41	82.0 (68.6, 91.4)	100.0 (92.9, 100)
**GenAI no**	9	50	59		

## Discussion

The current iterations of GenAI systems are more flexible and user-friendly than older models, both likely catalysts for their relatively rapid adoption.^6^ Despite these promising features, a major and ongoing challenge for the integration of GenAI systems in health care is the need for advanced computing infrastructure to process data and apply these computationally complex models. Training and deployment of GenAI systems require substantial computational resources and time due to the complexity and size of these models.^10,15^ To address these barriers to support scaling on a larger scale, we first applied an algorithm using less computationally intensive strategies to flag CIED procedures with >10% probability of CIED infection to reduce the size of the cohort to a more manageable size for processing and application of the GenAI and then provided filtered unstructured clinical data with iteratively developed prompts to support the second phase of the review process for identifying true CIED infections (Figure 1). We then used GenAI to replicate the data abstraction steps of the manual review process and to summarize the information to streamline review for CIED infections. Overall, we found that the GenAI augmented process had outstanding specificity and good sensitivity; the most common source of errors related to the GenAI’s ability to correctly sequence events and to determine attribution, similar to the errors identified during a multisite deployment of GenAI to support CLABSI surveillance.^16^

HAIs are reported as a quality metric, and given the limitations of currently available GenAI tools, manual adjudication and review of output continues to be an important step for ensuring appropriate attribution, classification, and reporting of cases, particularly for SSI, which, unlike CAUTI and CLABSI, include clinician diagnosis as one of the surveillance criteria.^14,17,18^ A persistent source of error for GenAI tools continues to be the creation of timelines of events, an essential element for determining attribution of possible HAIs. Some, but not all, of these errors can be addressed with iterative prompt updates, but it remains unclear if and when systems will evolve beyond AI-assisted review to fully automated processes. Additionally, manual reviews often provide critical insights for root cause analysis and patient safety interventions, and thus, even if the process could be fully automated, human review is likely to continue to be necessary to use the surveillance data to inform patient safety interventions. Despite these limitations of currently available tools and challenges with scaling, most systems lack mechanisms for comprehensive surveillance of CIED infections and other patient safety events.^18^ Thus, even imperfectly accurate systems offer a framework for expanding these services beyond traditional inpatient settings to encompass a broader array of clinical care.

A major benefit of the GenAI-assisted process was its ability to rapidly process, review, and organize information from a large volume of clinical notes to facilitate manual determination. In our study, the GenAI-assisted process reduced case review time to 5–7 minutes per chart compared to manual chart reviews, which typically required at least 20 minutes per chart, with complex charts requiring up to 25 minutes for a thorough review. Additionally, the GenAI system offers the opportunity for a “second check” on the manual review process, which is also imperfect, with prior work finding that corrections are required in up to 37% of cases with a single reviewer.^19^ A structured chart review conducted by GenAI, followed by a brief secondary review by an expert, may be an approach that balances accuracy with human resource requirements.^19,20^

One of the most high-profile concerns about GenAI systems is their propensity to “hallucinate” or to provide answers that are not grounded in the data. The GenAI deployed in most healthcare systems, including VA GPT, is set to a temperature of zero to minimize this risk, and prior evaluations suggest that errors of omission may be more common than errors of commission for summarization tasks performed in the healthcare setting.^21,22^ Although our dataset was limited, we reassuringly did not find any evidence of hallucinations in our study.

Our strategy of first limiting the cohort using less computationally intensive strategies and then using the GenAI on the more limited dataset reduces but does not eliminate concerns about the costs associated with the computing and scaling of these tools. Text-note searching tools, such as VA Voogle, a web-based application available in the VA, is a keyword-based search engine that can flag notes with specific terms but does not have organization or summarization capabilities. GenAI supports interactive querying, allowing users to ask follow-up questions and receive refined answers in real time. The system is also able to adjust its responses based on the evolving context of queries, but these systems can present information in an overly confident manner even when determinations are incorrect and have a tendency to be sycophantic. Ultimately, both chart-review support tools have limitations. An additional consideration for future improvement would be to use a search tool such as Voogle to identify the most important clinical notes for review followed by a GenAI-assisted process to support higher-level tasks, such as determination of whether an infection was present at the time of admission and/or attributed to a specific procedure; this would partially replicate the filtering and note selection process applied in this study. Other model types may be better suited for tasks where timelines are important for determining attribution and application of these models should be an area of future investigation.^23^

### Limitations

This study was performed within the VA Healthcare System, and while it included Community Care data as part of the flagging process, the findings primarily demonstrate the feasibility and effectiveness of natural language processing in a national healthcare setting specific to VA as we did not have access to clinical note data from outside the VA for the purposes of this study. Results may not be directly generalizable to other healthcare systems. The analysis involved data filtering prior to input into the GenAI tool, which is likely to increase the apparent accuracy of the tool by directing the model to the most relevant data. We did not attempt to directly apply the GenAI tool to an unfiltered dataset. However, given prior work suggesting high specificity of these tools, applying the GenAI to less complex cases may increase apparent accuracy.

The precise impact of these steps on the performance of GenAI performance remains unclear, although there may be bias toward improved accuracy relative to real-world performance or performance at scale. Future studies need to evaluate the method’s efficacy without extensive data filtering to understand its robustness and applicability in raw data contexts. Our application of the GenAI tool was limited to a specific subset of clinical notes, and the inclusion of more complex and potentially contradictory clinical data may impact performance. Additionally, we did not perform a comparative analysis of time saved by using our proposed GPT-4o tool against other established tools such as Voogle, and it is possible that other less computationally complex strategies could yield similar time savings.

## Conclusions

HAI surveillance activities are a promising application of GenAI tools, such as VA GPT 4.0, with the potential to streamline the manual review process and save time. Careful attention is needed to ensure appropriate attribution and timing of HAI events. Filtering strategies to reduce the number of cases that use the computationally resource-intensive GenAI tools can enhance feasibility and support scaling. A hybrid informatics approach using machine learning and structured data, followed by GenAI, may facilitate the expansion of HAI surveillance activities.

## Supporting information

Basnet et al. supplementary material Basnet et al. supplementary material

## Supplementary material

The supplementary material for this article can be found at https://doi.org/10.1017/ice.2025.10384.
