# Supplementary material for: Leveraging a large language model to support expansion of surveillance activities to include cardiovascular implantable device infections in a large, integrated national healthcare system
Source: Infect Control Hosp Epidemiol. 2026 Jan 23;47(4):351–6. doi: 10.1017/ice.2025.10384 (PMC13216803; doi:10.1017/ice.2025.10384)
Supplement: Basnet et al. supplementary material [file S0899823X2510384Xsup001.docx]

Supplementary Material 1 : Manual Review Tool for CIED Chart Abstraction

**Procedure Details**

1. Patient SSN
2. Procedure date

Enter the date flagged from CDW for review

1. Station/Sta3N
2. Procedure type Initial procedure placement

(Initial, revision, upgrade) Revision - battery replacement Revision - device upgrade


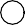

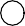

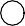

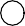


Procedure to manage complication (e.g., hematoma, infection, lead dislodgement)

1. Original device insertion date (approximate)

Note: If exact date is not available, enter 01-01-YYYY

with the appropriate year. (MM-DD-YYYY)

1. If the procedure was performed for a complication, what was the stated complication?
2. Additional procedure notes

**CIED Infection Details**

1. Any CIED infection present? Yes


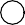

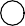


No

1. Date of CIED infection

10 Was the device infected at the time of procedure? Yes No

(Check yes for this value if the procedure was for MANAGEMENT of an existing device infection ONLY. If the provider does not mention in the notes that the device was infected at the time of the procedure, select no).


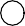

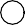


1. Did the CIED infection occur within 90 days of a Yes device procedure? No
2. Are the Specified Index Procedure Date and Infection Yes (index procedure date is correct) Date the same? No (infection from a different procedure)

(In other words, was the procedure at the SAME time/date the infection was diagnosed?)


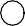

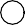

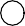

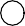


1. What is the correct procedure date associated with the patient's CIED infection?
2. If Infection present at the time of the procedure, what was the original procedure date?
3. What is the suspected source of the original Procedure infection? UTI/urinary tract

Skin/soft tissue


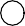

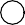

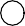

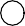

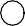

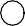

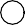


In other words, where does the treating physician Diabetic foot infection/chronic foot wound think the infection originated? Some infections are Dental procedure/odontogenic

caused by device contamination at the time of the GI

device procedure (either original placement or Other (specify) revision) and some are caused by other infections that

travel through the bloodstream and then "stick" to the device. Infections can start in other places in the

body (e.g., urinary tract, skin, diabetic foot ulcer) and then travel and infect the device.

1. Infection Source Notes. Please use this field for free text entry/notes that are helpful for

re-review/clarification.

1. Infection Source- Snips from EHR
2. Check the relevant CIED Infection Criteria:
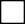
 Clinician Diagnosis of Infection (with treatment)


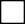
 Erythema/redness

(Check all that apply)
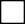
 Tenderness/pain at insertion site


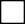
 Pus or drainage from the insertion site If using symptoms based criteria, then at least two
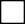
 Swelling at insertion site

must be present in order to determine whether an
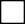
 Fever

infection is present or not. E.g., Pain and swelling
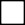
 Other systemic signs of infection (chills, sweats) without other symptoms or a clinician diagnosis of
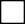
 Echocardiographic evidence of infection (lead hematoma should not be counted as an infection without involvement or valve involvement/endocarditis) other criteria present.
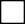
 Positive wound or blood cultures

1. Type of CIED Infection. Cellulitis only

Pocket infection


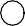

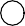

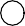

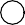

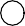

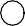


Cellulitis only = skin involvement only, no deeper Endocarditis

infection of the device. Lead infection

Stitch abscess Other (specify)

Pocket infection = infection of the pocket (e.g., pus/infected battery) but no evidence of systemic infection (e.g., bloodstream infection) or infection of the deeper part of the device (like the leads).

Endocarditis = Infection of the heart valves, with or without lead involvement (typically diagnosed via Duke's Criteria with a strong emphasis on cardiac imaging/echocardiogram).

Lead infection = Infection of the device leads (typically diagnosed via echocardiogram/cardiac imaging and/or removal of the device).

Stitch abscess = Pus at the site of a stitch (typically treated with stitch removal and no other treatment).

1. Other infection type
2. Additional infection notes
3. Documentation of infection (if needed) from EHR

**Infection Diagnosis Details**

1. CIED Infection Criteria - Microbiology. Yes No


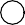

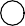


Was the infection diagnosed using microbiologic criteria (e.g., positive wound or blood culture)?

1. Positive wound or blood cultures
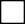
 Blood culture positive
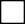
 Wound culture positive
2. Date of first positive blood culture
3. Blood culture positive results Staph aureus - MSSA Staph aureus - MRSA

Coagulase negative Staph aureus Streptococcal species Enterococcal species


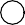

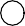

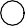

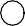

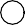

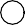

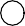

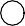

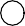


E. Coli Pseudomonas Klebsiella Other (specify)

1. Other positive blood culture
2. Date of first positive wound culture
3. Wound culture positive results Staph aureus - MSSA Staph aureus - MRSA

Coagulase negative Staph aureus Streptococcal species Enterococcal species


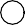

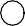

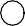

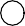

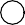

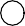

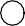

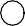

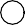


E. Coli Pseudomonas Klebsiella Other (specify)

1. Other positive wound culture

31 Additional diagnosis notes

1. Infection Diagnosis SNIP from EHR


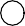

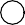


**Treatment Details**

1. CIED Infection Intervention No Intervention Pocket/abscess drainage


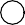

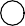

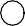

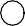

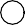


Did the patient receive a procedure (or intervention) Removal of battery/pocket intervention only to manage the infection (e.g., in addition to Removal of entire device including leads

medical/antimicrobial treatment, did the patient also Attempted removal of entire device including leads receive a procedural or surgical intervention)? but materials retained

1. Antibiotic treatment
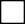
 Vancomycin


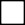
 Daptomycin What antibiotics were used to treat the infection (can
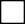
 Cefazolin select more than one, as these may change over the
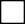
 Cefepime course of the patient's treatment as more is learned
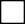
 Ceftriaxone

about the causative organism)?
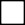
 Ampicillin


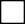
 Ampicillin/sulbactam
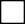
Piperacillin/tazobactam
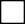
 Linezolid


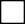
 Bactrim (trimethoprim/sulfamethoxazole)
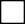
 Doxycycline


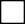
 Minocycline


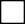
 Augmentin (amoxicillin/clavulanate)
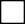
 Amoxicillin


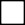
 Keflex (cephalexin)
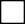
 Dicloxacillin


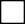
 Rifampin


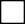
 Other (specify)

1. Other Antibiotic Treatment

If the antibiotic selected for treatment is "other," please specify the type here.

1. If rifampin was given, was the device retained? Yes No
2. Additional treatment notes
3. Infection Treatment SNIP
4. Requires Second Review Yes

No


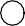

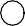


1. Notes for second review

**Supplementary Material 2: Utilized GenAI Prompt for CIED Infection Determination**

**Instructions:**

1. **Summarize Patient Case:**
   - **Task:** I have provided information about a patient. Summarize the case including the *date of* *Cardiac Implantable Electronic Device (CIED) procedure, type of CIED procedure done, admission diagnosis, and discharge notes.* DO NOT include the patient’s name, personally identifiable information, or any other private identifiers.
2. **Review for CIED Infection:**
   - You are reviewing a patient’s chart to determine if they have a true Cardiac Implantable Electronic Device (CIED) Infection. Below is the summary of the guidelines:

**Guidelines for CIED Infections:**

- **Definition and Classification:**
  - **CIED Infection:**
    - **Definition:** A CIED infection refers to an infection that occurs in patients who have cardiac implantable electronic devices such as pacemakers, implantable cardioverter-defibrillators (ICDs), or cardiac resynchronization therapy (CRT) devices. These infections can involve different parts of the device:
      - **Device Pocket:** The area where the device is implanted under the skin.
      - **Leads:** The wires that connect the device to the heart.
      - **Both Pocket and Leads:** Infections can involve both the pocket and the leads.
    - **Types of Infections:**
      - **Cellulitis only:** Infection limited to the skin around the device, without deeper involvement.
      - **Pocket infection:** Infection of the device pocket, which may present with pus or other signs of infection but without systemic infection.
      - **Endocarditis:** Infection of the heart valves, which may or may not involve the device leads. Typically diagnosed using Duke's Criteria with a focus on cardiac imaging.
      - **Lead infection:** Infection of the device leads, often diagnosed through echocardiography or during device removal.
- **CIED Infection Criteria:**
  - At least two of the following symptoms must be present to diagnose an infection: erythema, tenderness, pus, swelling, fever, systemic signs, echocardiographic evidence, positive cultures.
  - **Deep Infections:**
    - **Definition:** Deep infections extend beyond the superficial tissues and may involve the device pocket, leads, or systemic involvement.
    - **Signs and Symptoms:** Persistent or worsening pain, significant swelling, purulent drainage, device erosion, systemic manifestations such as fever, chills, malaise, and clear signs of bacteremia or endocarditis.
- **Comprehensive Microbiology:**
  - Positive blood or wound cultures indicating bacteria or microorganisms. Common pathogens include MSSA, MRSA, CoNS, Pseudomonas, Klebsiella, Enterococcal Species, E. coli, Gram-negative bacteremia.
- **Diagnostic Approaches:**
  - **Echocardiography:** Findings supporting true CIED infection.

**Prompt for Diagnosing CIED Infection:**

1. **Date of CIED Infection:**
   - **Context:** You are provided with clinical notes regarding a patient who has a Cardiac Implantable Electronic Device (CIED). Your task is to accurately identify the date of the CIED infection. Consider infection-related keywords: Focus on mentions of infection-related terms such as "infection," "erythema," "tenderness," "pus," "swelling," "fever," "systemic signs," and results from diagnostic tests like echocardiography or culture reports. If multiple dates are mentioned, select the date most directly associated with the onset or diagnosis of the infection symptoms. The infection date is the earlier of either the initial presentation date or the first positive microbiologic culture date. What is the most accurate date corresponding to the CIED infection based on the clinical notes?
   - Was this the initial device implant?
   - What was the date of the implant?
   - What was the type of CIED procedure? (e.g., pacemaker, ICD, CRT)
   - Was the infection within the 90-day CIED surveillance window date?
   - Is the flagged procedure date and infection date the same? If No, what is the correct procedure date related to the infection?
   - State the type of CIED infection
2. **Confirmed CIED Infection:**
   - Did the patient have a confirmed CIED infection?
3. **Diagnostic Criteria:**
   - What were the criteria used to diagnose the infection? (e.g., erythema, tenderness, pus, swelling, fever, systemic signs, echocardiographic evidence, positive cultures)
4. **Comprehensive Microbiology:**

Provide details on any positive blood culture and/or wound culture, including the specific pathogens identified (e.g., MSSA, MRSA, CoNS, Pseudomonas, Klebsiella, Enterococcal Species, E. coli, Gram-negative bacteremia).

**Blood Culture:**

- - Was the blood culture positive on admission?
  - If yes, what were the results of the positive blood culture? (e.g., pathogens present such as S. aureus, CoNS, E. coli, etc.)

1. **Other Positive Cultures:**
   - Were there any other positive cultures? (e.g., wound culture)
   - If yes, what were the results of the positive wound culture? (e.g., pathogens present such as S. aureus, CoNS, E. coli, etc.)
2. **Source of Bacteremia:**
   - What was the most likely source of bacteremia?
3. **Systemic Infection Signs:**
   - Were there any signs of systemic infection? (e.g., TTE or TEE findings) Echocardiographic evidence of infection (lead involvement or valve involvement or endocarditis)
4. **Device Removal:**
   - Did the patient undergo complete device removal/system explant?
5. **Detailed Clinical Signs and Symptoms:**
   - Describe any localized erythema, minimal swelling, warmth, mild tenderness, or minimal purulence confined to the incision.
   - Describe any persistent or worsening pain, significant swelling, purulent drainage, device erosion, systemic manifestations (fever, chills, malaise), and clear signs of bacteremia or endocarditis.
6. **Non-True Infection Indicators:**
   - Were there any signs or symptoms indicative of non-true infections? (e.g., pain and swelling without other symptoms, clinician diagnosis of hematoma without other criteria)
7. **Additional Diagnostic Tests:**
   - Were any additional diagnostic tests performed to confirm the infection? (e.g., imaging studies such as TTE or TEE, additional cultures)
8. **Treatment:**
   - What treatment was administered for the infection? (e.g., antibiotics, surgical intervention)
9. **Patient History:**
   - Does the patient have a history of previous CIED infections or other relevant medical conditions that could influence the diagnosis?
10. **Follow-Up:**
    - What follow-up measures were taken to monitor the patient’s condition post-treatment?

**Supplementary Material 3: Illustrative Case Output Data generated by GenAI for CIED Infection Determination**

Summary of Patient Case:

The patient, an XX year old male with a history of atrial fibrillation, hypertension, asthma, peripheral vascular disease, hyperlipidemia, diabetes mellitus type II, and heart failure with reduced ejection fraction, underwent an upgrade of a previous pacemaker to a cardiac resynchronization therapy defibrillator (CRT-D) on XX. He was admitted for suspected syncope and decompensated congestive heart failure (D-CHF). During his hospital stay, he developed low back pain, worsening leukocytosis, and increased inflammatory markers. On XX blood cultures revealed Enterococcus faecalis, and a Gallium scan showed L2-L3 intervertebral space discitis. An echocardiogram indicated mitral valve endocarditis, which was confirmed by transesophageal echocardiography (TEE) showing vegetations on the mitral valve and the right ventricular lead. Despite being treated with Ampicillin and ceftriaxone since XX the device was not removed due to ventricular pacing dependence. Following a comprehensive discussion with the family, the patient was placed under hospice care. He was monitored until he passed away on XX. The infection was confirmed based on positive blood cultures, echocardiographic evidence, and systemic signs of infection such as fever and chills. The most likely source of bacteremia was the infected cardiac device leads and mitral valve endocarditis.

Review for CIED Infection:

Date of CIED Infection:

The infection was identified on XX based on the positive blood cultures and Gallium scan results indicating an infection involving the cardiac device leads and the mitral valve.

Was this the initial device implant?

No, it was an upgrade of a previous pacemaker to a CRT-D.

What was the date of the implant?

XX

What was the type of CIED procedure?

Cardioverter-defibrillator implantation with left ventricular lead insertion (CRT-D).

Was the infection within the 90-day CIED surveillance window date?

Yes, the infection was diagnosed within 90 days of the implant (XX).

Is the flagged procedure date and infection date the same?

No, the infection date is XX, while the procedure date was YY.

State the type of CIED infection:

Endocarditis involving the mitral valve and pacemaker lead infection.

Confirmed CIED Infection:

Yes, the patient had a confirmed CIED infection.

Diagnostic Criteria:

Positive blood cultures, echocardiographic evidence of vegetations on the mitral valve and the right ventricular lead, systemic signs of infection (fever, chills, leukocytosis, increased inflammatory markers).

Blood Culture:

Yes, the blood culture was positive upon admission.

Results: Enterococcus faecalis

Other Positive Cultures:

Not mentioned specifically, hence no additional positive wound cultures indicated.

Source of Bacteremia:

The most likely source of bacteremia was the infected cardiac device leads and mitral valve endocarditis.

Systemic Infection Signs:

Yes, echocardiographic evidence showed vegetations on the mitral valve and right ventricular lead, along with systemic signs such as fever and chills.

Device Removal:

No, the device was not removed due to ventricular pacing dependence.

Detailed Clinical Signs and Symptoms:

Symptoms included persistent low back pain, significant swelling, increased inflammatory markers, worsening leukocytosis, purulent drainage, and vegetations confirmed by TEE, indicating endocarditis.

Non-True Infection Indicators:

No signs indicating non-true infections were reported.

Comprehensive Microbiology:

Positive blood cultures identified Enterococcus faecalis. The detailed microbiology of any wound cultures was not reported.

Additional Diagnostic Tests:

TEE (Transesophageal Echocardiography) confirmed vegetations on the mitral valve and pacemaker lead.

Gallium scan indicated L2-L3 discitis.

Treatment:

The patient was treated with antibiotics, specifically Ampicillin and ceftriaxone. Hospice care with comfort measures was provided later due to the poor prognosis.

Follow-Up:

The patient transitioned to hospice care and was monitored until he passed away on XX.
